# CLU, FOS, and CXCL8 as diagnostic biomarkers for heart failure progression post-acute myocardial infarction: an integrated RNA-Seq and multi-machine learning study

**DOI:** 10.3389/fcvm.2025.1611668

**Published:** 2025-06-30

**Authors:** Jingjing Wei, Peng Yu, Yucai Hu, Lijie Qiao, Bin Li, Haitao Li, Shiyang Xie, Zhengwei Dong, Aolong Wang, Yilin Zhang, Xinlu Wang, Yongxia Wang, Mingjun Zhu

**Affiliations:** ^1^ Heart Center, The First Affiliated Hospital of Henan University of Chinese Medicine, Zhengzhou, China; ^2^ Department of Orthopedics, The First Affiliated Hospital of Henan University of Chinese Medicine, Zhengzhou, China; ^3^ Henan Provincial Research Center for Traditional Chinese Medicine Orthopedics and Traumatology Specialty Technology and Equipment Engineering, The First Affiliated Hospital of Henan University of Chinese Medicine, Zhengzhou, China; ^4^ Henan Evidence-based Medicine Center of Chinese Medicine, The First Affiliated Hospital of Henan University of Chinese Medicine, Zhengzhou, China

**Keywords:** heart failure, acute myocardial infarction, biomarker, immune infiltration, bioinformatics analysis

## Abstract

**Background:**

Heart failure (HF) secondary to acute myocardial infarction (AMI) remains a public health concern. Peripheral blood mononuclear cells (PBMCs) are the essential initiators of heart failure after myocardial infarction (HFpAMI). We aimed to identify PBMCs-related critical genes as diagnostic biomarkers for HFpAMI and analyze the immune infiltration patterns.

**Methods:**

Differential expression genes (DEGs) from PBMCs microarray data of AMI with or without HF were identified. Functional enrichment analysis was used to explore the biological roles of DEGs. Subsequently, candidate biomarkers were identified using machine learning and the MCODE plugin, with ROC used to describe the accuracy. CIBERSORT was utilized to investigate immune infiltration. Multi-level validation of our findings was conducted, including RNA-seq profiling of the external cohort, RT-qPCR, and flow cytometry analyses on PBMCs samples.

**Results:**

In the comparison between 30 HFpAMI and 34 non-HF samples, 27 DEGs were identified. Functional enrichment analysis suggested that DEGs may be involved in the pathological process of HFpAMI by participating in immune-inflammatory response. Employing machine learning and MCODE assessment, we identified three robust potential biomarkers (CLU, FOS, and CXCL8). Immunological analysis revealed a marked increase in neutrophils and decrease in CD4T cells. In the external validation cohort, RNA-seq analysis demonstrated consistent upregulation of CLU, FOS, and CXCL8 in HFpAMI compared to non-HF controls. RT-qPCR and flow cytometry further corroborated these expression trends and their correlations with neutrophil infiltration, CD4T cells and M2 macrophage concentration reductio, aligning with bioinformatics predictions. ROC analysis validated the diagnostic efficacy of these biomarkers, with CLU exhibiting the highest AUC (0.833, 95% CI: 0.679–0.988), followed by FOS (0.809, 95% CI: 0.64–0.977) and CXCL8 (0.802, 95% CI: 0.635–0.970).

**Conclusions:**

Significantly upregulated DEGs, including CLU, FOS, and CXCL8, might be served as novel diagnostic biomarkers for HFpAMI, and dysregulated immune infiltration hinted possible the immune system intervention point in the setting of HFpAMI.

## 1 Introduction

Heart failure (HF) is acknowledged as a global epidemic, impacting around 64.34 million individuals globally, leading to a 5-year mortality rate as high as 50% (1). A comprehensive study on heart failure incidence and prevalence in 2021 revealed a standardized prevalence of 1.10% among the Chinese population aged 25 and above, estimating a total of 12.1 million cases (2). Each year, almost 3 million new cases arose, resulting in an average of 3.3 hospitalizations, and incurring an average annual hospitalization cost of $4,406.8 per person, thereby placing a substantial economic burden on public health. Acute myocardial infarction (AMI), a severe form of coronary heart disease, was a primary contributor to HF (3). A growing body of evidence suggested that approximately 40%–56% of patients experienced a decline in cardiac function after AMI, with about 25%–33% of patients progressing to HF (4, 5). The rehospitalization rate was two times higher, and the mortality rate was four times higher for these patients compared to those without AMI. Therefore, early diagnosis is crucial for reducing the incidence of HF in AMI patients at high risk of HF progression. Identifying early biomarkers associated with heart failure after myocardial infarction (HFpAMI) may help address this issue. Although several biomarkers such as natriuretic peptides, cardiac troponin, Galectin-3, and soluble suppression of tumorigenicity-2 (sST2) have been proposed for the diagnosis and risk assessment of HFpAMI, their effectiveness in early prediction remains limited. For example, the specificity of natriuretic peptides may be affected by confounding factors such as renal dysfunction and aging (6). Cardiac troponin, despite its value in detecting myocardial injury, provides limited sensitivity in predicting the transition to heart failure (7). Similarly, while Galectin-3 and sST2 are associated with fibrosis and inflammation, their prognostic performance has been inconsistent across different patient populations (8). These challenges suggest the need to explore more reliable and mechanistically relevant biomarkers that reflect key pathological processes such as immune and inflammatory dysregulation, in order to improve early identification and risk stratification of patients susceptible to heart failure after acute myocardial infarction.

Following myocardial infarction, apoptotic and necrotic myocardial cells release damage-associated molecular pattern proteins, activating the innate immune system and triggering a severe inflammatory response. Prolonged and excessive activation of the inflammatory response can lead to an expansion of the injury area, exacerbating tissue damage, ultimately resulting in HF (9). Peripheral blood mononuclear cells (PBMCs) originate from bone marrow hematopoietic stem cells and are closely related to the occurrence and development of cardiac remodeling after AMI. They swiftly migrate into the bloodstream from the bone marrow and spleen within hours of myocardial injury, infiltrating the infarcted region and contributing to the inflammatory immune response (10). Therefore, genes implicated in monocyte/macrophage recruitment for cardiac remodeling may serve as potential biomarkers for early identification of HF progression risk among AMI patients.

**Figure 1:**
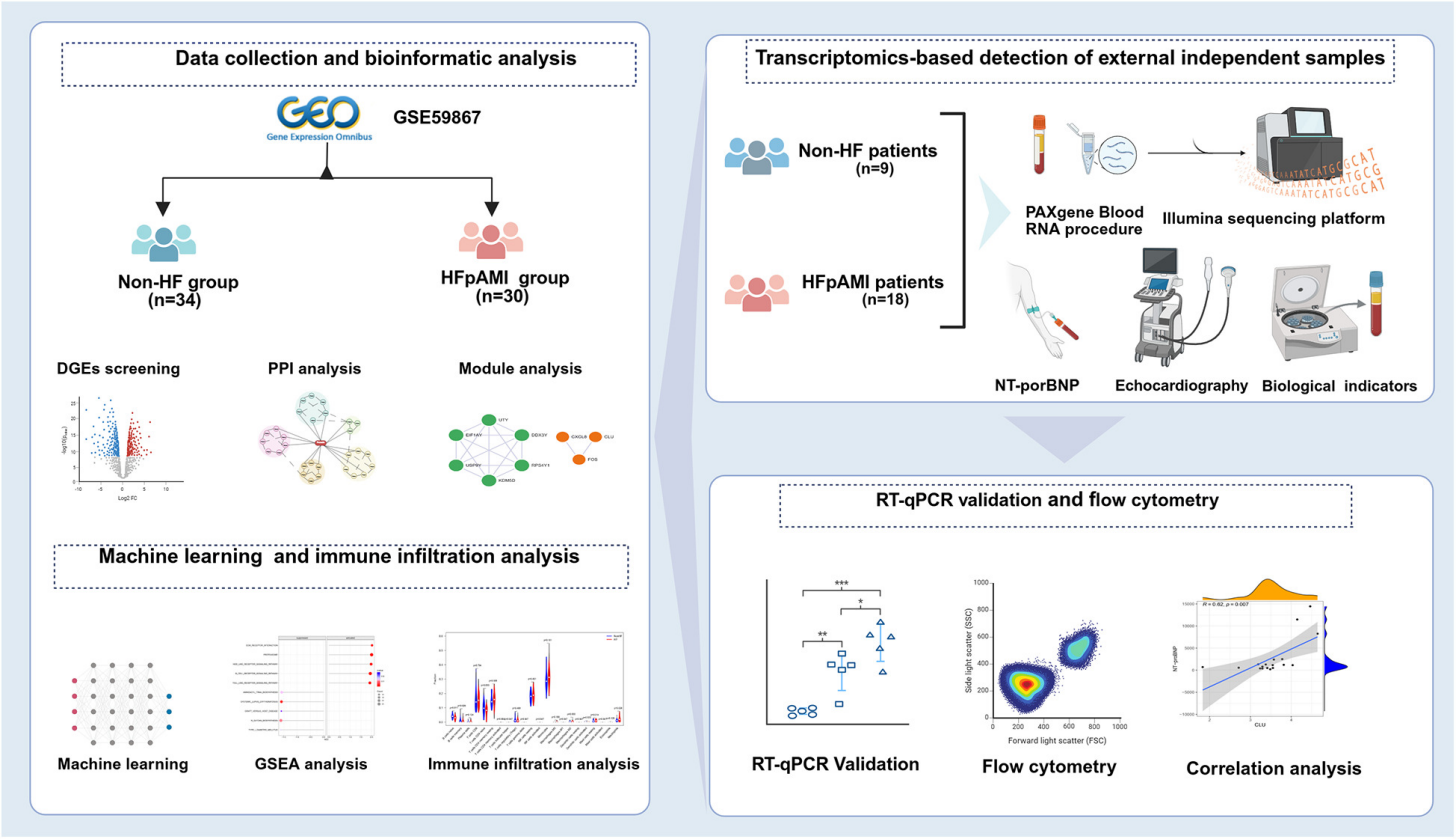
The flowchart of integrated bioinformatics and machine learning to identify predictive biomarkers of HFpAMI.

High-throughput next-generation transcriptome sequencing offers an unbiased and comprehensive overview of gene expression features in disease models, serving as a novel and practical approach for screening specific biomarkers in cardiovascular disease. Nevertheless, differential expression genes (DEGs) detected in transcriptome analysis may exhibit limitations in reproducibility and sensitivity. Machine learning algorithms can improve the prediction and accuracy of identifying DEGs using traditional microarray or next-generation sequencing data, confirming potential biomarkers or disease diagnostic features (11). Commonly utilized machine learning techniques comprise the least absolute shrinkage and selection operator (LASSO) regression, support vector machine recursive feature elimination (SVM-RFE), and random forest (RF) algorithms. In recent years, CIBERSORT algorithm, as a widely applied analytical method, has been frequently used to study immune cell infiltration patterns in diseases based on transcriptomic sequencing or microarray data and to evaluate the infiltration proportions of 22 immune cells in each sample (12). However, there has been no study combining machine learning algorithms with CIBERSORT to identify peripheral monocyte-associated genes of HFpAMI (13, 14). Therefore, in the present study, we conducted an analysis of the GSE59867 dataset from various perspectives. Initially, using the “limma” R package and Gene Ontology (GO) and Kyoto Encyclopedia of Genes and Genomes (KEGG) pathway enrichment analysis, we identified DEGs and critical pathways involved in the progression to HF after AMI at the resolution of PBMCs. Subsequently, we systematically screened and identified diagnostic biomarkers related to HFpAMI using three machine learning methods and molecular complex detection (MCODE) algorithms. Moreover, CIBERSORT was utilized to assess immune cell infiltration patterns in HFpAMI, followed by a comprehensive analysis of the correlation between candidate biomarkers and immune cells. To further validate the robustness of our findings, we collected an independent cohort of HFpAMI patients and AMI patients without HF, and performed RNA sequencing (RNA-seq) on these external samples. Finally, we validated the expression levels of the selected candidate biomarkers and explored their role in the mechanism of HFpAMI (Figure 1).

## 2 Methods

### 2.1 Data collection and quality control

Utilizing the GEO database (https://www.ncbi.nlm.nih.gov/geo/), we conducted a search using “human,” “myocardial infarction,” and “heart failure” as filtering criteria, ultimately acquiring the GSE59867 dataset. This dataset was generated using the Affymetrix Human Gene 1.0 ST Array (GPL6244), covering 28,869 well-annotated genes. Raw microarray data underwent rigorous quality control: missing values were imputed via the *k*-nearest neighbors algorithm (*k* = 10) using the *impute* R package and samples with >20% missing probes were excluded. Probe-level filtering was performed with the *arrayQualityMetrics* R package, removing probes with intensity values below the 10th percentile or detection *P*-value >0.05. Probes were mapped to the Ensembl GRCh38 genome annotations to ensure accuracy. The final dataset included 64 PBMCs samples from AMI patients with or without HF development during a 6-month follow-up (HFpAMI, *n* = 30 vs. non-HF, *n* = 34).

### 2.2 DEGs screening and functional enrichment analysis

The GSE59867 dataset underwent normalization using the impute.knn function from the “impute” R package and the normalizeBetweenArrays function from the “limma” package. Filtering values were set at |logFC| > 0.585 and *p*-value < 0.05 to obtain the DEGs from the GSE59867 dataset. R package “Clusterprofiler” and “DOSE” were used to perform GO, and KEGG enrichment analysis.

### 2.3 Protein-protein interaction (PPI) network construction and module analysis

Mapping DEGs to the STRNG database (https://string-db.org/), with a filter criterion of a minimum required interaction score of 0.4, removing unconnected nodes to establish the PPI network. After downloading PPI analysis results, we input them into the cytoscape software (version 3.10.2) for further optimization. The MCODE plugin computes a score for each node in the network based on the connections around the node and the average connections of all nodes. It expands relevant nodes based on these scores to form clusters. We proceeded to perform cluster analysis on the PPI network using the MCODE plugin, configuring the parameters as follows: node degree threshold ≥2, haircut ≥0.2, node score threshold ≥0.2, K-core ≥2, maximum depth = 100, in order to select significant functional clustering modules within the network.

### 2.4 Screening candidate biomarkers using machine learning techniques

In this study, the LASSO analysis was performed using the “glmnet” function in the R programming language to generate a sequence of regularization parameters. Each regularization parameter value underwent tenfold cross-validation (*k* = 10). Following cross-validation, the regularization parameter associated with the lowest cross-validation error was chosen as the optimal parameter. The LASSO model was applied to identify differential feature genes (15). The SVM-RFE can handle high-dimensional data and nonlinear problems, showing robustness that enhances algorithm accuracy. Through the SVM-RFE algorithm, implemented using the “e1071” package in the R programming language, we conducted randomized feature extension on the data to choose the best feature subset. We employed cross-validation methods to assess and optimize the SVM model, selecting the most optimal differential genes. The RF algorithm, known for its robustness, flexibility, and high accuracy, was utilized in this study. Using the “random Forest” package in the R programming language, the RF algorithm conducted random sampling of data and built decision tree models. Following this, an evaluation of the RF model was performed to select significant differential feature genes. Ultimately, the intersection of differential feature genes identified by the three machine learning methods was obtained to determine the candidate biomarkers associated with the disease, and the receiver operating characteristic (ROC) curves were plotted.

### 2.5 Gene set enrichment analysis (GSEA)

The exploration of potential biological markers' functions and biological processes was conducted using GSEA. Based on the median expression levels of potential biological markers, they were divided into high and low expression groups. GSEA using the KEGG signaling pathway genes as the preset gene set was performed to explore the functions and biological processes of potential biological markers in the dataset.

### 2.6 Immune infiltration analysis

The R package “CIBERSORT” was employed to quantify the relative proportions of 22 immune cell subtypes in PBMCs samples using the LM22 signature matrix (version 1.1), a validated gene expression reference containing 547 marker genes for 22 human hematopoietic cell types, including naive B cells, memory B cells, plasma cells, CD8+ T cells, CD4+ T cells (naive, memory resting, activated), regulatory T cells (Tregs), γδ T cells, natural killer cells (resting/activated), monocytes, macrophages (M0/M1/M2), dendritic cells (resting/activated), mast cells (resting/activated), eosinophils, and neutrophils. Raw gene expression data were normalized using the “voom” method to adjust for sequencing depth and variance. CIBERSORT analysis was performed with 1,000 permutations to ensure robust deconvolution accuracy, and only samples with a CIBERSORT *P* < 0.05 were retained for subsequent analyses. Differential immune infiltration between HFpAMI and non-HF groups was assessed via the Mann–Whitney *U* test. Spearman correlation analysis (two-tailed, *P* < 0.05) was then applied to explore associations between candidate biomarkers and immune cell subtypes.

### 2.7 Independent clinical validation samples

From June 2022 to July 2023, 18 HFpAMI patients (HFpAMI group) and 9 AMI patients without HF (non-HF group) were recruited from the First Affiliated Hospital of Henan University of Chinese Medicine. HFpAMI was defined as new-onset heart failure occurring within 6 months after AMI diagnosis. All patients had a history of coronary angiography and infarct-related revascularization treatment. Pharmacological treatment was administered according to the current guidelines. All participants provided written informed consent prior to the commencement of the study. This research protocol was approved by the Ethics Committee of the First Affiliated Hospital of Henan University of Chinese Medicine (20211HL-178) and conducted in accordance with the Helsinki Declaration.

### 2.8 Diagnostic criteria

The diagnostic criteria for AMI followed the Chinese Society of Cardiology's 2019 “Diagnosis and Treatment Guidelines for Acute ST-Segment Elevation Myocardial Infarction,” while the diagnostic criteria for HF were in accordance with the “Chinese Guidelines for the Diagnosis and Treatment of Heart Failure 2018” (16, 17).

### 2.9 Inclusion criteria

(1) age range of 40–80 years; (2) subjects meeting diagnostic criteria for AMI and HF; (3) duration of disease within 6 months.

### 2.10 Exclusion criteria

(1) active inflammation; (2) patients with other potential cardiac diseases (such as severe valve abnormalities, myocardial disease, or congenital heart disease); (3) patients with liver and/or kidney dysfunction, tumors, and autoimmune diseases.

### 2.11 Clinical indicators measurement

NT-proBNP was measured using a colloidal gold assay kit (GeteinBiotech, Nanjing, China). Left ventricular ejection fraction (LVEF), and left ventricular end diastolic dimension (LVEDD) were determined using a color Doppler ultrasound diagnostic device (GE Vivid E95, USA) with the two-dimensional ultrasound Simpson's method. Neutrophil count was assessed using the XN1000 fully automated hematology analyzer (SYSMEX, Japan).

### 2.12 RNA-seq-based transcriptomic study

#### 2.12.1 Sample collection and RNA extraction

Fasting venous blood samples (2.5 ml) were collected from each subject in the morning using PAXgeneTM Blood RNA Tubes (PreAnalytiX, Cat. No. 765165, China). After gentle inversion (8–10 times for mixing), samples were labeled and stored at −80°C following the manufacturer's protocol. Total RNA was extracted using the PAXgene Blood miRNA Kit (PreAnalytiX, Cat. No. 765444, China) with on-column DNase I digestion to remove genomic DNA contamination. RNA concentration was quantified using a Nanodrop spectrophotometer (BioForge, China), and RNA integrity was assessed via the Agilent 5400 Bioanalyzer (Agilent Technologies, USA) with RNA Integrity Number (RIN) ≥7.0 as the inclusion criterion. Purity was confirmed by A260/A280 ratios (1.8–2.1) and A260/A230 ratios (>1.5). Potential DNA contamination was excluded by agarose gel electrophoresis (1% gel, 120 V, 20 min).

#### 2.12.2 Library construction and sequencing

Total RNA (1 μg per sample) was used as input. mRNA was enriched using poly-T oligo-attached magnetic beads [NEBNext Poly(A) mRNA Magnetic Isolation Module, Cat. No. E7490, USA]. The stranded RNA-seq library was constructed using the NEBNext Ultra II RNA Library Prep Kit (Cat. No. E7770, USA) following the manufacturer' s protocol. Briefly, mRNA was fragmented to ∼250 bp, followed by first-strand cDNA synthesis with random hexamers and reverse transcriptase. Second-strand cDNA was synthesized using dUTP to preserve strand specificity. After end repair, adenylation, and ligation of indexed adapters, cDNA fragments of 300–400 bp were selected using AMPure XP beads (Beckman Coulter, USA). PCR amplification was performed with 12 cycles. Library quality was verified using the Agilent 5,400 Bioanalyzer (DNA High Sensitivity Kit). Paired-end sequencing (2 × 150 bp) was performed on the Illumina NovaSeq 6,000 platform (Illumina, USA) with an average sequencing depth of 50 million reads per sample. HISAT2 v2.0.5 was employed to construct the reference genome index and align paired-end clean reads to the reference genome. Read counts for each gene were quantified using featureCounts (v1.5.0-p3). FPKM (Fragments Per Kilobase of exon model per Million mapped reads) values were then calculated for each gene, normalizing for gene length.

#### 2.12.3 DEGs screening and functional enrichment analysis

Gene counts were normalized using DESeq2 (v1.20.0), and fold changes (FC) were calculated. *P*-values were adjusted using the Benjamini-Hochberg method to control the false discovery rate. DEGs were identified using the criteria of |log2FC| ≥ 1 and adjusted *P* < 0.05. Functional enrichment analysis of DEGs was performed using the R packages “ClusterProfiler” (v4.0.5) and “DOSE” (v3.18.1) for GO and KEGG pathways.

### 2.13 PBMCs isolation, RNA extraction, and reverse transcription

The density gradient centrifugation using Ficoll Paque Plus (Cytiva, USA) was conducted following the prescribed procedure to isolate PBMCs from 10 ml of EDTA-containing whole blood (12). RNA extraction from PBMCs was performed using TRIzol reagent (GeneCopoeia, USA) as per the manufacturer's instructions, and RNA concentration was measured using a nanodrop spectrophotometer. Reverse transcription was carried out using the Surescript™ First-Strand cDNA Synthesis Kit (GeneCopoeia, USA) at 37°C for 60 min, followed by incubation at 85°C for 5 min, and the resulting cDNA was stored at −80°C.

### 2.14 Real-time quantitative polymerase chain reaction (RT-qPCR)

The RT-qPCR was conducted using BlazeTaq™ SYBR® Green qPCR mix 2.0 (GeneCopoeia Green, USA), with primer sequences listed in Table 1. A housekeeping gene (GAPDH) was used as an endogenous control for normalization. The Relative mRNA expression was calculated using the 2^−ΔΔCt^ method in a triplicated manner.

**Table 1:** Primer information.

Gene		Primer
CLU	Forward	CTACTTCTGGATGAATGGTGACC
	Reverse	CGGGTGAAGAACCTGTCCT
FOS	Forward	TCCAAGTGCCGAAAAAGGAAG
	Reverse	CGAGTTCTGAGCTTTCAAGGT
CXCL8	Forward	ACTGAGAGTGATTGAGAGTGGAC
	Reverse	AACCCTCTGCACCCAGTTTTC
TLR4	Forward	AGACCTGTCCCTGAACCCTAT
	Reverse	CGATGGACTTCTAAACCAGCCA
MYD88	Forward	GGCTGCTCTCAACATGCGA
	Reverse	CTGTGTCCGCACGTTCAAGA
GAPDH	Forward	CCATGGGTGGAATCATATTGGA
	Reverse	TCAACGGATTTGGTCGTATTGG

### 2.15 Flow cytometry

Removed the PBMCs samples from −80°C storage, rapidly thawed them at 37°C, and then diluted them with preheated culture medium, adding 500 ul of PBS (Solarbio, Beijing, China) to resuspend the cells to a density of 1–2 × 106/ml. The cell suspension was collected afterward. Subsequently, the cells were resuspended in 1 ml of staining buffer (Elabsience, Wuhan, China), centrifuged at 300 × g for 5 min, repeating this step twice. Then, 5 ul of Anti-Human CD45 Antibody (Elabsience, Wuhan, China), Anti-Human CD3 Antibody (Elabsience, Wuhan, China), Anti-Human CD4 Antibody (Elabsience, Wuhan, China), and Anti-Human CD8a Antibody (Elabsience, Wuhan, China) were added to the resuspended cells according to the antibody instructions and incubated at room temperature, avoiding light, for 15 min. After washing, the cells were resuspended in 300 μl of staining buffer and finally analyzed using the FACSCelesta flow cytometer (BD, USA). A similar methodology was used for macrophage typing, wherein 5 ul of Anti-Human CD11b Antibody (Elabsience, Wuhan, China), and Anti-Human CD86 Antibody (Elabsience, Wuhan, China) were added to the resuspended cells. Following the wash, it was important to add 250 μl of 1X Cytofix/Cytoperm Buffer (BD, USA) to the resuspended cells and incubate them at room temperature, avoiding light, for 20 min. Subsequently, the fixed-permeabilized cells were resuspended, Anti-Human CD206 Antibody (Elabsience, Wuhan, China) was added, and they were incubated at room temperature while avoiding light for 30 min. Following this, the cells were resuspended using Permeabilization Wash Buffer (BD, USA) and analyzed using a flow cytometer.

### 2.16 Statistical analysis

The statistical analysis was designed and executed by the data administrator from the Henan Evidence-based Medicine Center of Chinese Medicine. All statistical analyses were performed using R statistical software (version 4.3.1, R Foundation for Statistical Computing, Vienna, Austria). Categorical data were presented as frequencies and percentages. Quantitative data were assessed for normality using the Shapiro–Wilk test and expressed as mean ± standard deviation (SD). Depending on the normality of the data, either Student's *t*-test or Mann–Whitney *U* test was utilized to compare continuous data between groups. Spearman's rank correlation coefficient was employed for correlation analysis. ROC curves were generated to assess the diagnostic capability of candidate biomarkers for HFpAMI. All statistical analysis tests were conducted using two-sided hypothesis tests. *P* < 0.05 was considered to be statistically significant for the differences tested.

## 3 Results

### 3.1 Identification of HFpAMI-related DEGs in GSE59867

**Figure 2:**
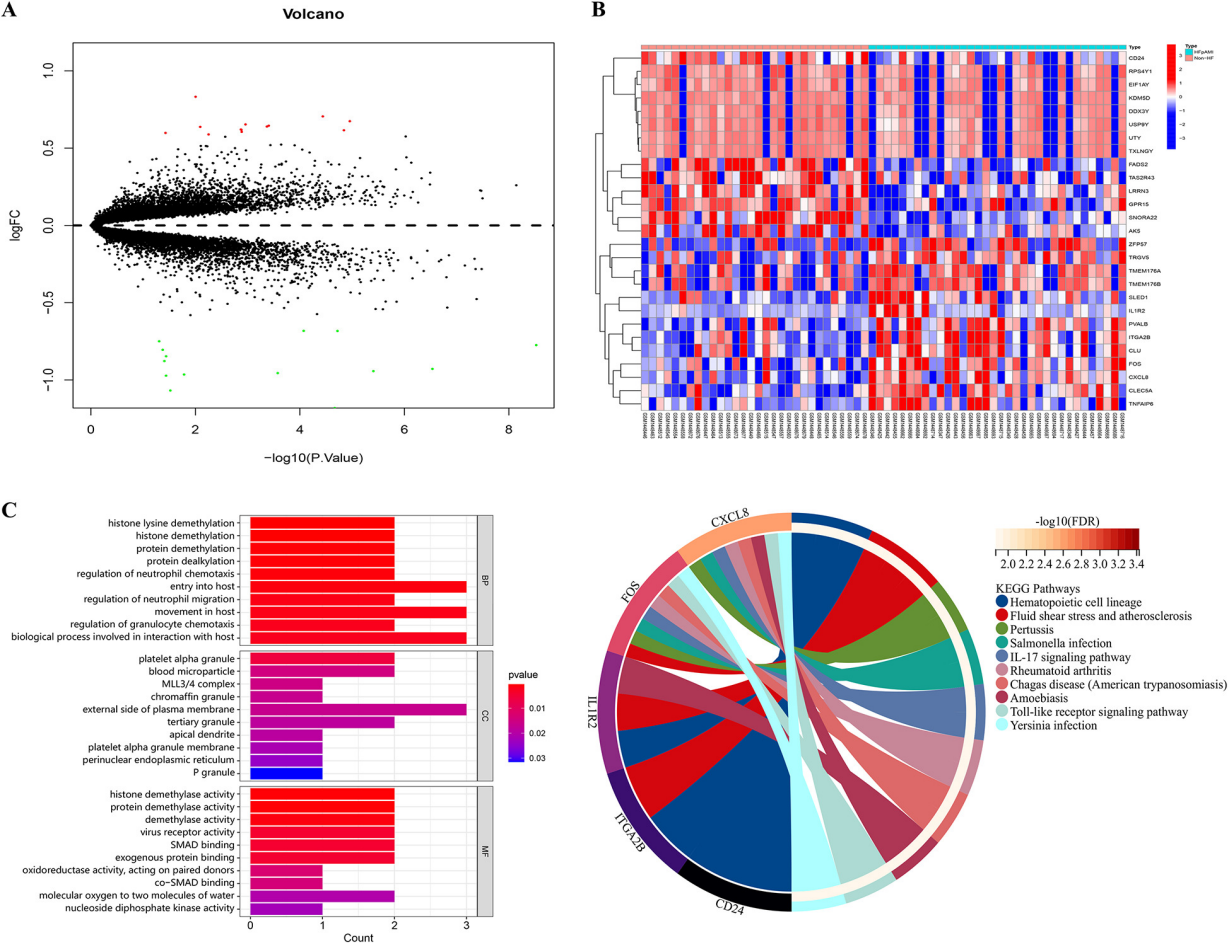
Volcano plot, heatmap and functional enrichment analysis of DEGs. **(A)** The volcano plot of DEGs. Red: upregulated genes; Green: downregulated genes; **(B)** the clustered heatmap showed the expression profiles of DEGs across different samples; **(C)** GO enrichment analysis; **(D)** KEGG pathway analysis.

We downloaded the microarray expression dataset from the GEO database and conducted differential expression analysis based on the aforementioned selection criteria. Consequently, we identified 27 DEGs in the GSE59867 dataset, with 13 downregulated and 14 upregulated expressions (Supplementary Table S1). Figure 2A illustrates the volcano plot of DEGs, while Figure 2B presents the clustered heatmap showing the expression profiles of DEGs across different samples.

### 3.2 Functional enrichment analysis of DEGs

For elucidating the biological functions and pathways associated with HFpAMI-related DEGs, we conducted GO functional annotation and KEGG pathway analysis. The GO enrichment analysis unveiled the involvement of DEGs primarily in biological processes concerning protein demethylation and neutrophil migration. In terms of cellular components, DEGs exhibited strong associations with blood micro-particles, platelet alpha granules, and the MLL3/4 complex. Molecular functions emphasized significant enrichment of DEGs in activities such as protein demethylase, protein binding, and oxidoreductase activities (Figure 2C). The KEGG pathway analysis demonstrated that the DGEs were primarily enriched in pathways related to atherosclerosis, and immune inflammatory responses, including the Toll-like receptor (TLR) signaling pathway and IL-17 signaling pathway (Figure 2D). Based on these enrichment results, DEGs may impact the pathogenesis of HFpAMI by regulating protein demethylation, kinases, and immune cell inflammatory responses.

### 3.3 PPI network and candidate biomarkers selection

**Figure 3:**
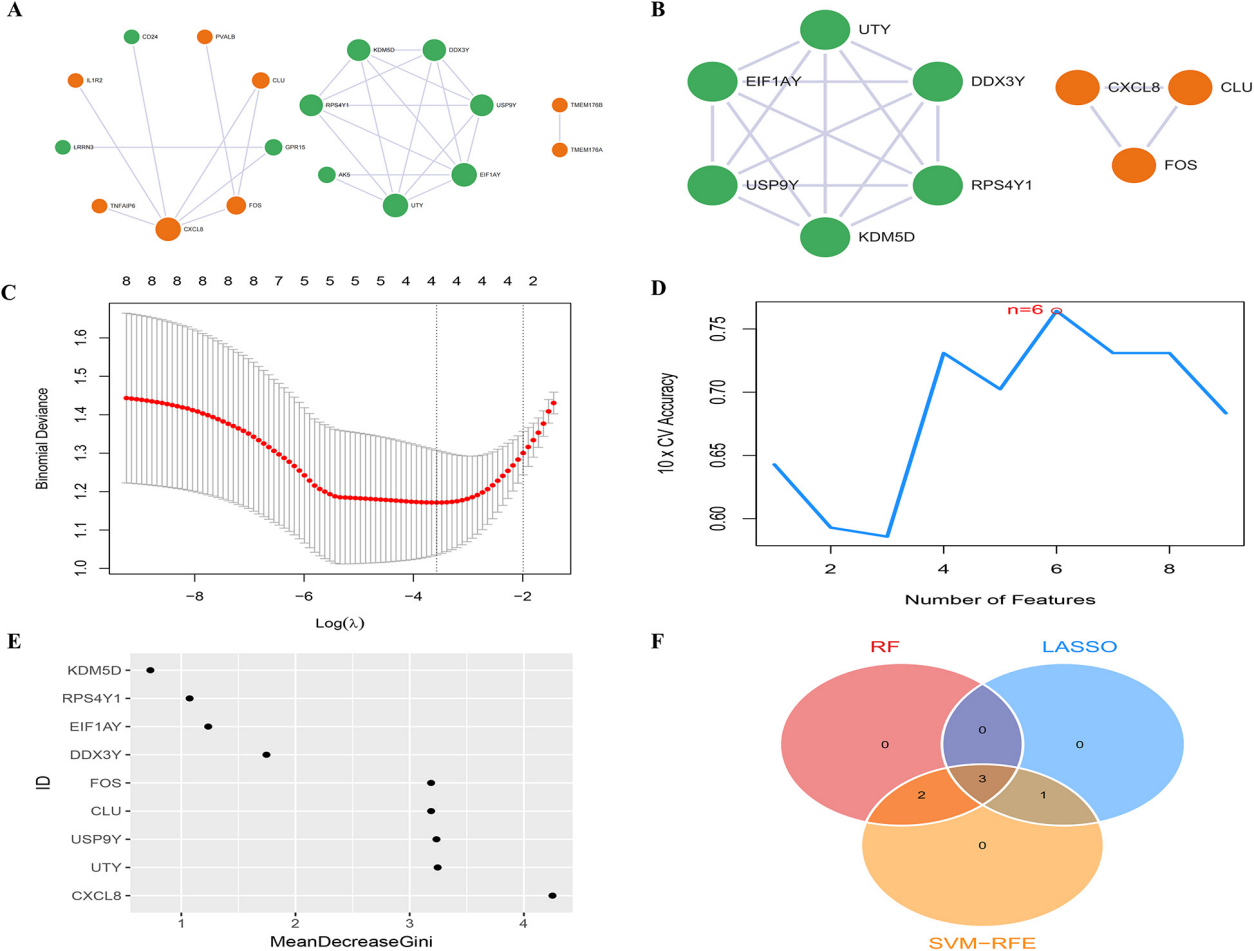
PPI network and candidate biomarkers selection. **(A)** The PPI network between DEGs has 18 nodes and 25 edges, the color of the nodes reflects up-regulated or down-regulated gene expression, with orange representing up-regulated expression and green representing down-regulated expression. The size of the node reflects the degree value, and the larger the node, the higher the degree value; **(B)** the PPI network of 2 high-scoring module genes based on Cytoscape plug-in MCODE analysis; **(C)** LASSO analysis screening of feature genes; **(D)** SVM-RFE algorithm screening of feature genes; **(E)** RF algorithm screening of feature genes; **(F)** Venn diagram of the intersection of diagnostic markers obtained by the three algorithms.

The PPI network of DEGs, constructed and refined using Cytoscape, consisted of 3 subnetworks, comprising a total of 18 nodes and 27 edges (Figure 3A, Supplementary Table S2). Following modular evaluation using the MCODE plugin, two crucial clustered functional modules were identified within this network (Figure 3B). Functional Module 1 did not exhibit any pertinent biological functions, while Functional Module 2 (CLU, FOS, and CXCL8) was primarily associated with immune inflammatory response functions.

**Figure 4:**
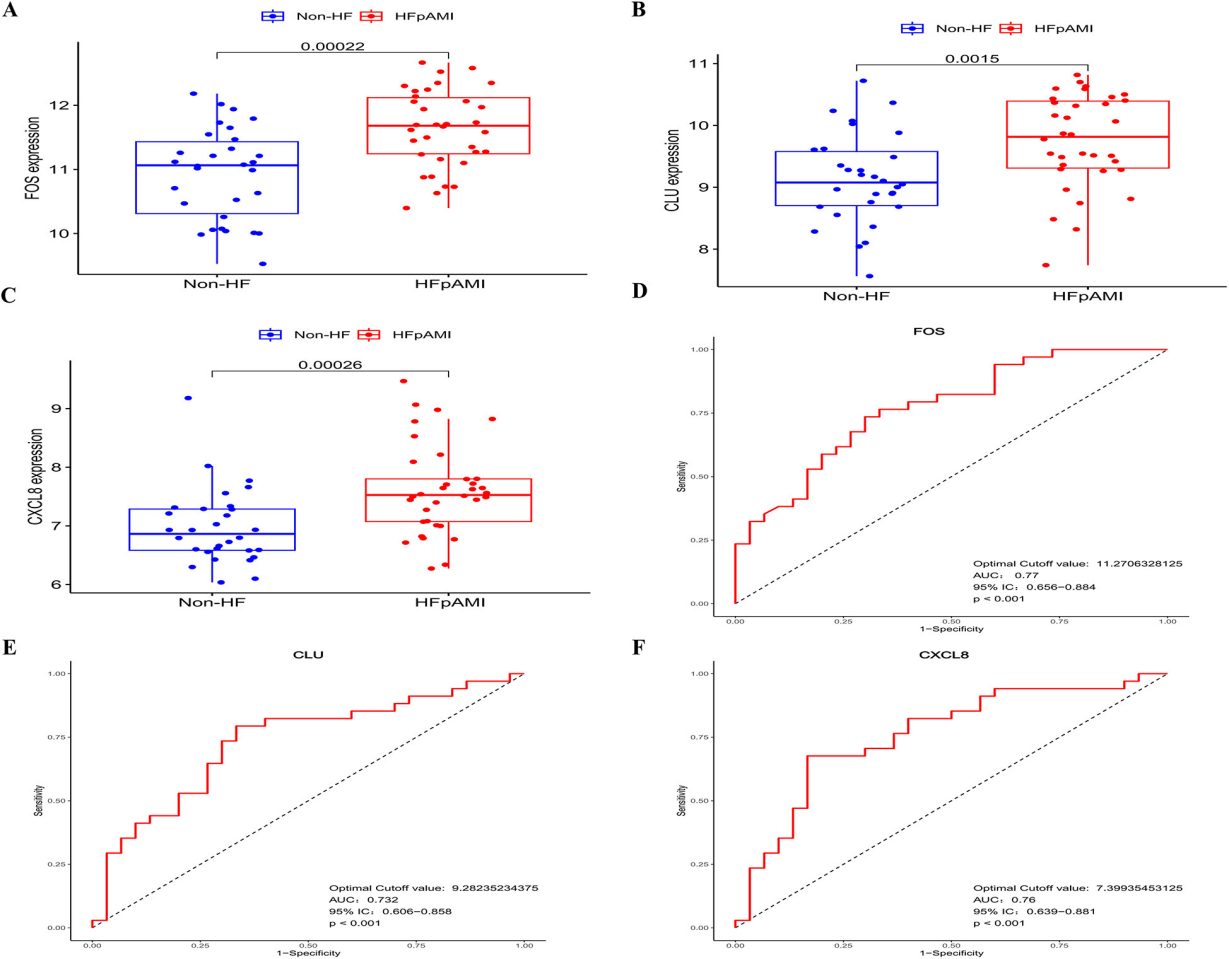
The expression levels and ROC curves of FOS, CLU, and CXCL8 in GSE59867. **(A)** The expression of FOS in GSE59867; **(B)** the expression of CLU in GSE59867; **(C)** The expression of CXCL8 in GSE59867; **(D)** ROC curve of FOS; **(E)** ROC curve of CLU; **(F)** ROC curve of CXCL8.

In the LASSO analysis, the four DEGs exhibiting the least bias in the binomial distribution model were identified as disease feature genes: FOS, CLU, CXCL8, and EIF1AY (Figure 3C). Meanwhile, the SVM-RFE algorithm identified six DEGs with the highest accuracy in 10-fold cross-validation as disease feature genes: USP9Y, UTY, CLU, FOS, EIF1AY, and CXCL8 (Figure 3D). Additionally, the RF algorithm selected DEGs with MeanDecreaseGini values >3 as disease feature genes: CXCL8, UTY, USP9Y, CLU, and FOS (Figure 3E). The intersection of the three algorithms derived differential feature genes was presented in a Venn diagram, yielding the disease candidate biomarkers CLU, FOS, and CXCL8 (Figure 3F), which aligned with the significant clustering module 2 obtained through the MCODE plugin. Furthermore, statistical analysis was conducted on the expression levels of these candidate biomarkers among different groups within the dataset. The expression levels of all three candidate biomarkers were significantly elevated in the HFpAMI group compared to non-HF group (*P <* 0.05) (Figures 4A–C). ROC analysis of the candidate biomarkers revealed area under the curve (AUC) area under the curve (AUC) values of 0.770, 0.732, and 0.760 for FOS, CLU, and CXCL8, respectively (Figures 4D–F), indicating the accuracy and reliability of the selected candidate biomarkers. These findings underscored the high diagnostic value of the candidate biomarkers for HFpAMI, indicating their potential as relevant biological indicators.

### 3.4 GSEA of candidate biomarkers

**Figure 5:**
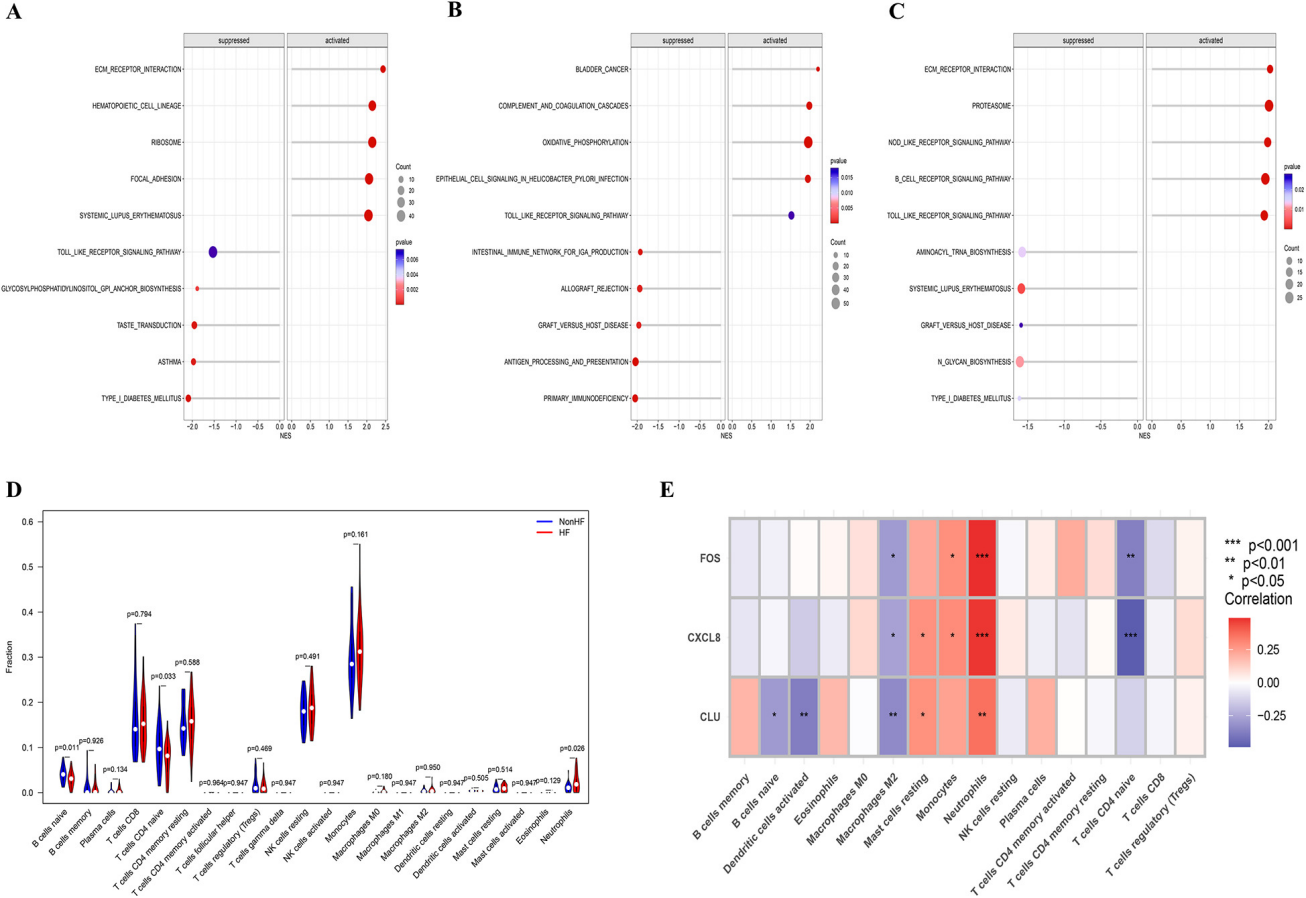
GSEA of candidate biomarkers and analysis of immune cell infiltration. **(A)** Biological pathways involved in CLU by GSEA; **(B)** biological pathways involved in FOS by GSEA; **(C)** biological pathways involved in CXCL8 by GSEA; **(D)** violin plot showing the comparison of 22 kinds of immune cells between HF and non-HF groups; **(E)** the heatmap of correlation between immune infiltrating cells and candidate biomarkers.

Further exploration was conducted on the specific signaling pathways associated with the candidate biomarkers and their potential molecular mechanisms affecting HF progression. GSEA results indicated the activation of CLU in ECM receptor interaction, hematopoietic cell lineage, and ribosome pathways, while its inhibition in TLR signaling, taste transduction, and asthma (Figure 5A); FOS activation was observed in complement and coagulation cascades, oxidative phosphorylation, and TLR signaling, with suppression in intestinal immune network for IgA and primary immunodeficiency pathways (Figure 5B); CXCL8 activation occurred in cellular signaling, gene expression, NLR receptor signaling, and TLR signaling, while being suppressed in Aminoacyl trna biosynthesis, and N-glycan biosynthesis, and systemic lupus erythematosus pathways (Figure 5C). These findings suggested that these candidate biomarkers may impact the occurrence and development of post-myocardial infarction HF through the modulation of immune-inflammatory responses. Furthermore, KEGG enrichment analysis of DEGs demonstrated significant enrichment of the TLR signaling pathway, which aligns with the prominent enrichment of the same pathway in the GSEA of candidate biomarkers. Consequently, this study postulated the pivotal role of this signaling pathway in the pathological process of post-myocardial infarction HF and suggests it as a critical pathway for further exploration of the pathological mechanisms of HFpAMI.

### 3.5 Results of immune cell infiltration

Through studying the relationship between immune infiltration and feature genes in the dataset, we explored the potential molecular mechanisms by which feature genes affect the progression of HFpAMI. Based on the results of immune cell infiltration (Figure 5D), the HFpAMI group exhibited significantly lower levels of immune infiltration for naive CD4T cells and naive B cells compared to the non-HF group (*P* < 0.05). Conversely, the immune infiltration level of neutrophils in the HFpAMI group was notably higher than that in the non-HF group (*P* < 0.05) (Figure 5D). Moreover, an exploration was conducted into the relationship between feature genes and immune cells. The findings revealed that CLU was inversely correlated with naive B cells and activated dendritic cells; CLU and CXCL8 showed a positive correlation with resting mast cells, while FOS and CXCL8 exhibited a positive correlation with monocytes and a negative correlation with naive CD4T cells. Particularly, FOS, CLU, and CXCL8 were concurrently negatively correlated with M2 macrophages and positively correlated with neutrophils (Figure 5E). The aforementioned outcomes showed the close association between feature genes and the infiltration levels of immune cells, demonstrating their pivotal role within the immune microenvironment.

### 3.6 Characteristics of the external clinical cohort

The study population comprised 18 patients with heart failure post-acute myocardial infarction (age: 64.39 ± 7.75) and 9 patients without heart failure (age: 61.44 ± 8.8), all meeting the inclusion criteria. Table 2 summarizes baseline demographic and cardiac function parameters. No significant differences were observed between the groups in age, gender, BMI, heart rate, or blood pressure (*P* > 0.05). All participants were of Han ethnicity. Notably, the HFpAMI group exhibited significantly higher NT-ProBNP and LVEDD values, and lower LVEF compared to the non-HF group. These findings indicate compromised cardiac structure and function in the HFpAMI group, consistent with the diagnostic criteria for HF.

**Table 2:** Baseline demographic and cardiac function parameters.

Parameters	HFpAMI group (*n* = 18)	Non-HF group (*n* = 9)	*P*-value
Age (years)	64.39 ± 7.75	61.44 ± 8.89	0.384
Female/male (*n*)	3/15	3/6	0.367
The Han nationality, *n* (%)	18 (100.00%)	9 (100.00%)	1
SBP (mmHg)	129.00 ± 22.17	127.78 ± 19.56	0.885
DBP (mmHg)	72.83 ± 12.72	74.33 ± 9.91	0.760
BMI, kg/cm^2^	23.64 ± 3.30	23.75 ± 2.62	0.937
HR, beat per minute	67.78 ± 10.02	66.78 ± 7.95	0.797
NT-ProBNP (pg/ml)	1,149.50 (498.75, 2,430.00)	114.67 (100.00, 125.50)	<0.001
LVEF (%)	39.17 ± 6.63	61.33 ± 3.77	<0.001
LVEDD (mm)	58.00 ± 5.17	47.33 ± 4.95	<0.001

Data are presented as mean ± SD or median (Q1, Q3) when appropriate.

SBP, systolic blood pressure; DBP, diastolic blood pressure; BMI, body mass index; HR, heart rate; NT-proBNP, N-terminal pro-B-type natriuretic peptide; LVEF, left ventricular ejection fraction; LVEDD, left ventricular end diastolic dimension.

### 3.7 Transcriptomic analysis of the external clinical cohort

**Figure 6:**
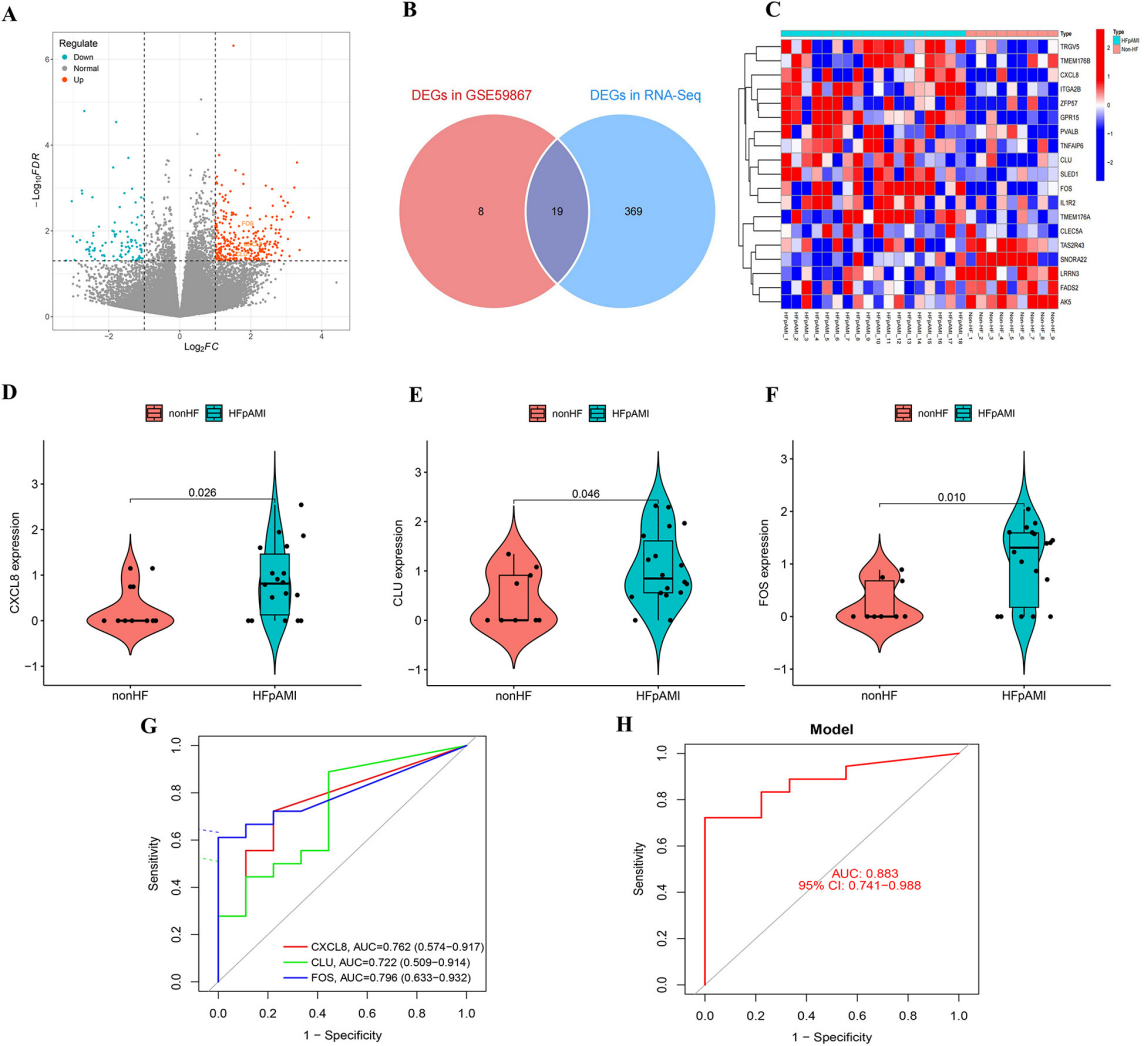
Transcriptomic analysis of the external clinical cohort. **(A)** Volcano map of transcriptome analysis; **(B)** venn diagram identifying 19 overlapping DEGs between GSE59867 and the external cohort; **(C)** heatmap demonstrating concordant expression patterns of the 19 shared DEGs; **(D)** the expression of CXCL8 in RNA-Seq; (**E**) the expression of CLU in RNA-Seq; **(F)** the expression of FOS in RNA-Seq; **(G)** ROC curve of CXCL8, CLU, and FOS; **(H)** combined diagnostic ROC curves of feature genes.

Transcriptome profiles of blood samples from the HFpAMI and non-HF groups were generated using the Illumina high-throughput sequencing platform. Differential expression analysis identified 388 DEGs (100 downregulated, 288 upregulated) between groups (Figure 6A). We performed intersection analysis between the 27 DEGs from the GSE59867 cohort and the 388 DEGs from our independent cohort. Venn diagram analysis revealed 19 overlapping DEGs (Figure 6B). Crucially, heatmap visualization demonstrated consistent expression patterns for these 19 genes across both cohorts (Figure 6C). Notably, the three candidate biomarkers CXCL8 (log2FC = 2.25, *P* = 0.02), CLU (log2FC = 1.60, *P* = 0.04), and FOS (log2FC = 2.36, *P* = 0.01) exhibited consistent upregulation in the HFpAMI group (Figures 6D–F). ROC analysis further confirmed their diagnostic potential, with AUC values of 0.762 (95% CI: 0.635–0.888) for CXCL8, 0.722 (0.579–0.865) for CLU, and 0.796 (0.673–0.919) for FOS (Figure 6G), the combined diagnostic performance of these biomarkers yielded an AUC of 0.883 (95% CI: 0.741–0.988) (Figure 6H). These results not only corroborated the findings from the GSE59867 dataset but also highlighted the robustness of CXCL8, CLU, and FOS as candidate biomarkers for HFpAMI.

### 3.8 Validation of the expression pattern of candidate biomarkers and immune cell infiltration

**Figure 7:**
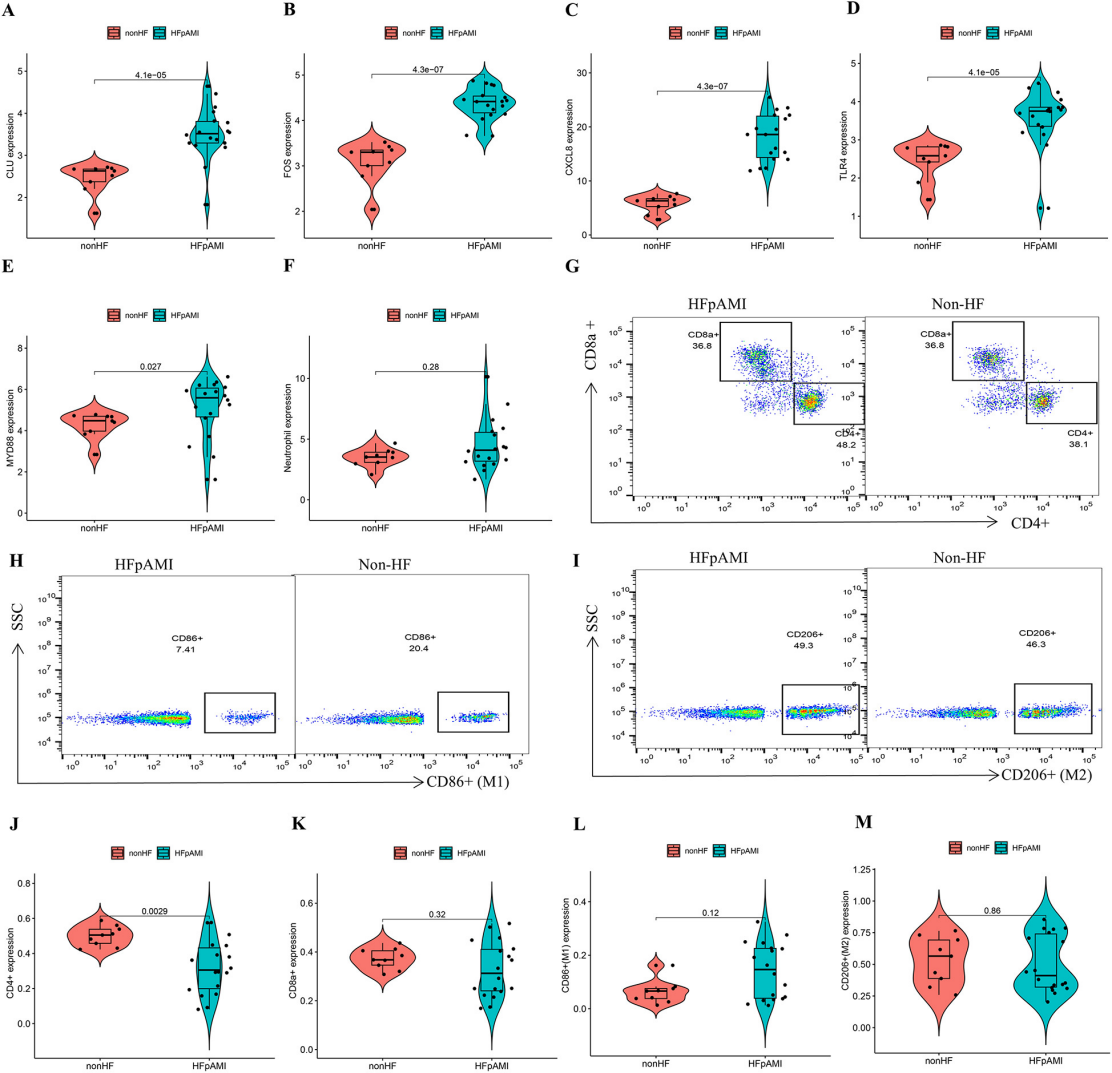
RT-qPCR, and flow cytometry analyses in the clinical samples. **(A)** The expression level of CLU; **(B)** the expression level of FOS; **(C)** the expression level of CXCL8; **(D)** the expression level of TLR4; **(E)** the expression level of MYD88; **(F)** the expression level of neutrophil count; **(G)** representative flow cytometry plots showing CD4T cells and CD8T cells; **(H)** representative flow cytometry plots showing M1 macrophages; **(I)** representative flow cytometry plots showing M2 macrophages; **(J)** expression of CD4T cells in different groups; **(K)** expression of CD8T cells in different groups; **(L)** expression of M1 macrophages in different groups; **(M)** expression of M2 macrophages in different groups.

**Figure 8:**
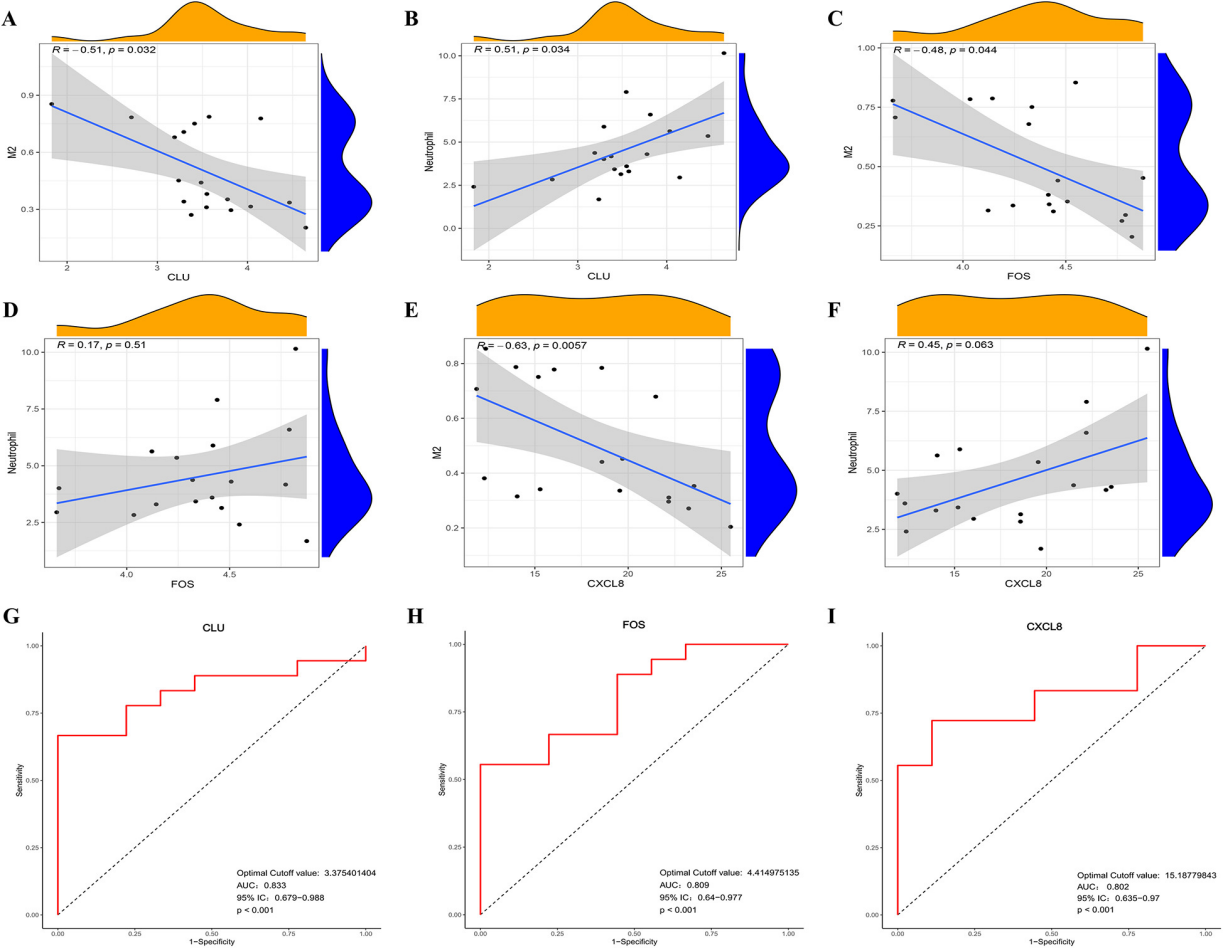
Correlation between the expression level of infiltrating immune cells and candidate biomarkers and ROC curve analysis in clinical samples. **(A)** Correlation between CLU and M2 macrophage expression; **(B)** correlation between CLU and neutrophil count; **(C)** correlation between FOS and M2 macrophage expression; **(D)** correlation between FOS and neutrophil count; **(E)** correlation between CLCL8 and M2 macrophage expression; **(F)** correlation between CLCL8 and neutrophil count; **(G)** ROC curve of CLU in clinical samples; **(H)** ROC curve of FOS in clinical samples; **(I)** ROC curve of CXCL8 in clinical samples.

The RT-qPCR results confirmed that the expression patterns of CLU, FOS, and CXCL8 were consistently upregulated in PBMCs samples of HFpAMI compared to non-HF group (*P* < 0.01) (Figures 7A–C), aligning with the main bioinformatics analysis. Moreover, we assessed key genes TLR4 and MYD88 in the TLR signaling pathway. The RT-qPCR results revealed elevated expression levels of TLR4 and MYD88 in the HFpAMI group compared to the non-HF group (*P* < 0.01) (Figures 7D,E), indicating the significant role of this signaling pathway in the pathophysiology of HFpAMI. Additionally, we measured the neutrophil count in patients. The results indicated a higher neutrophil count in the HFpAMI group compared to the non-HF group, although without statistical significance (Figure 7F) (*P* = 0.28). Furthermore, we validated immune infiltration and the correlation between feature genes and immune cells. CD4T cells, CD8T cells, M1 macrophages, and M2 macrophages could be detected in the PBMCs by flow cytometry. The findings indicated a significant decrease in CD4T cell concentration in the HFpAMI compared to the non-HF group (*P* < 0.01) (Figures 7G,J), with no significant differences observed in CD8T cells (Figures 7G,K), M1 macrophages (Figures 7H,L), and M2 macrophages (Figures 7I,M) between the two groups. Spearman correlation analysis unveiled a significant negative correlation between CLU and M2 macrophage concentration in patients with HFpAMI (*r* = −0.51, *p* = 0.032) (Figure 8A). CLU also showed a significant positive correlation with neutrophil count (*r* = 0.51, *p* = 0.034) (Figure 8B); Regarding FOS, a substantial negative correlation with M2 macrophage concentration was observed (*r* = −0.48, *p* = 0.044) (Figure 8C), along with a positive correlation with neutrophil count, although statistical significance was lacking (*r* = 0.17, *p* = 0.51) (Figure 8D); Similarly, CXCL8 exhibited a significant negative correlation with M2 macrophage concentration (*r* = −0.63, *p* = 0.0057) (Figure 8E) and a significant positive correlation with neutrophil count (*r* = 0.45, *p* = 0.063) (Figure 8F). These findings indicated an imbalance in certain types of immune cells during the progression of HFpAMI.

### 3.9 Verification of the candidate biomarkers for HFpAMI

**Figure 9:**
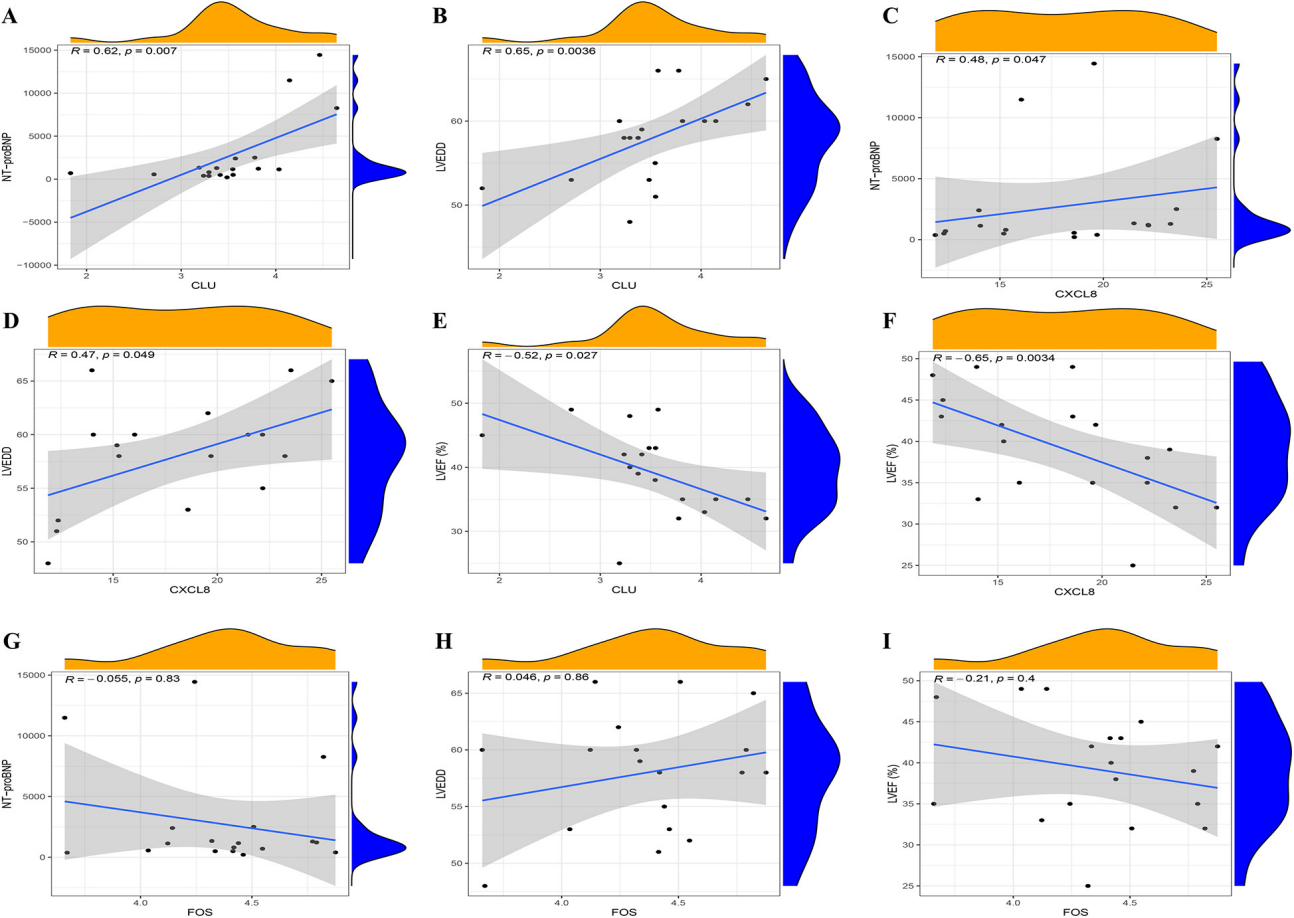
Spearman correlation analysis of candidate biomarkers and cardiac function indicators. **(A)** correlation analysis between CLU and NT-proBNP; **(B)** correlation analysis between CLU and LVEDD; **(C)** correlation analysis between CXCL8 and NT-proBNP; **(D)** correlation analysis between CXCL8 and LVEDD; **(E)** correlation analysis between CLU and LVEF; **(F)** correlation analysis between CXCL8 and LVEF; **(G)** correlation analysis between FOS and NT-proBNP; **(H)** correlation analysis between FOS and LVEDD; **(I)** correlation analysis between FOS and LVEF.

Furthermore, we conducted ROC curve analysis to verify the diagnostic value of the selected candidate biomarkers by assessing the gene expression levels between the HFpAMI and non-HF groups, as illustrated in Figures 8G–I. The AUC values for CLU, FOS, and CXCL8 were 0.833 [95% confidence interval (CI): 0.679–0.988], 0.809 (95% CI: 0.64–0.977), and 0.802 (95% CI: 0.635–0.970), respectively. These findings showed that these peripheral monocyte-associated genes are pivotal biomarkers for the progression of HFpAMI. Additionally, we investigated the correlation between CLU, FOS, and CXCL8 and cardiac function indicators to further evaluate diagnostic accuracy and reliability. The results demonstrated that CLU and CXCL8 had a strongly positive connection with NT-proBNP and LVEDD, with correlation coefficients between 0.47 and 0.65 (Figures 9A–D). Conversely, CLU and CXCL8 presented dramatically negative correlation with LVEF, with correlation coefficients of −0.65 and −0.52, respectively (Figures 9E,F). there was no observed correlation between FOS and cardiac function indicators (Figures 9G–I).

## 4 Discussion

AMI is widely recognized as a prevalent and significant contributor to HF worldwide.^3^ Although there have been improvements in the outcomes of HFpAMI due to the development of pharmaceutical and non-pharmaceutical treatments over the past few decades, the overall mortality rate, cardiovascular event occurrence, and readmission rates still remain discouraging. Many patients with HFpAMI remain undiagnosed in the early stages of the disease. Additionally, the lack of timely interventions or early preventive treatments in many patients is one of the major contributors to poor prognosis. Hence, there is an urgent need to identify and explore potential biomarkers for early screening and diagnosis of HFpAMI. Recent studies have demonstrated that combining high-throughput transcriptomics sequencing with various machine learning algorithms is an effective approach for identifying potential biomarkers and novel therapeutic targets in complex diseases (18, 19). To our knowledge, this study is the first to integrate multiple machine learning with the CIBERSORT algorithm and transcriptomics sequencing to identify and validate candidate diagnostic biomarkers for HFpAMI. This hybrid approach not only prioritizes robust biomarkers through consensus across three machine learning models but also contextualizes their roles within immune microenvironment dynamics—revealing their correlation with neutrophil infiltration and CD4+ T cell depletion, a dimension overlooked in prior biomarker studies. This approach provides new insights into the molecular mechanisms underlying the pathogenesis of HFpAMI.

In this study, a series of bioinformatics analyses were conducted to screen DEGs related to post-AMI HF and non-HF PBMCs in the GSE59867 dataset, yielding a total of 27 DEGs. To elucidate the biological functions and pathways associated with the DEGs, GO functional annotation and KEGG pathway analyses were conducted. GO functional annotation revealed that the biological processes and molecular functions of the DEGs primarily involve the regulation of interleukin secretion, granulocyte migration, chemokine activity, and CXCR chemokine receptor binding. KEGG pathway analysis revealed significant enrichment involving the TLR signaling pathway, Lipid and atherosclerosis and NF-kappa B signaling pathway. These findings indicated active involvement of DEGs in the process of immune inflammation. To identify potential diagnostic biomarkers for HFpAMI, we employed three machine learning methods, multiple algorithm evaluations using the CytoHubb plugin, and validation with the external independent clinical cohort. This comprehensive approach led to the identification of three robust potential biomarkers from the DEGs: CLU, FOS, and CXCL8. CLU, constitutively expressed in most mammalian tissues, is a highly conserved protein with various biological functions including regulation of complement activity, lipid transport, and inhibition of cell apoptosis. Normally, CLU expression levels are low but significantly elevate under stress-induced pathological conditions (20). Several studies have demonstrated that after AMI, patients exhibit increased CLU levels in the heart and blood, which were correlated with left ventricular remodeling (21). Although recent studies have suggested an independent association of CLU with the severity of the condition and the survival of patients with HF (22), it remains inconclusive whether CLU levels in PBMCs of post-AMI can serve as valuable biomarkers for determining HF progression. FOS can be expressed in cardiomyocytes, endothelial cells, and vascular smooth muscle cells, and its expression level is closely associated with the development and progression of various cardiovascular disease (23). The protein encoded by FOS can form a transcription factor complex AP-1 with Jun family proteins through a leucine zipper. In ischemic cardiomyopathy and dilated cardiomyopathy, the expression of AP-1 in cardiac tissue significantly increases (24). Recently, Zhuang L and colleagues reported that the abnormal activation of Fos/AP-1 transcriptional activity is associated with pro-inflammatory responses. Inhibiting Fos/AP-1 signal activity could effectively reduce ischemia-induced immune responses and produce therapeutic effects by alleviating adverse cardiac remodeling and HF (25). CXCL8, also known as IL-8, is a major mediator of inflammatory responses, primarily activating or attracting neutrophils. Previous studies have indicated that serum IL-8 levels in AMI patients after percutaneous intervention might serve as predictive markers of HF development (26). Here, our team reported for the first time the aberrant levels of CLU, FOS, and CXCL8 between PBMCs samples of post-AMI HF and non-HF. Furthermore, ROC analysis confirmed their high specificity and sensitivity in diagnosing HFpAMI. Importantly, in our cohort, our team validated that the expression levels of CLU, FOS, and CXCL8 in the HFpAMI group were significantly upregulated compared to non-HF group. Moreover, CLU and CXCL8 exhibited significant positive correlations with NT-proBNP and LVEDD, while demonstrating significant negative correlations with LVEF. Our research findings suggested that CLU, FOS, and CXCL8 serve as potential diagnostic biomarkers for HFpAMI.

Intriguingly, our GSEA targeting three potential diagnostic biomarkers showed a significant enrichment of the TLR signaling pathway, which was further substantiated by significant enrichment in the KEGG analysis of DEGs, suggesting the TLR signaling pathway as a crucial pathway in the progression of HFpAMI. TLRs function as primary receptors of the innate immune system, triggering innate immune defense through interactions with pro-inflammatory pathways, thus contributing to the onset and exacerbation of inflammatory diseases. Reports indicated that following myocardial infarction, necrotic myocardial cells and damaged extracellular matrix released danger signals (27). Throughout HF progression, various TLRs engaged across diverse cardiovascular tissues and cells, amplifying the inflammatory response. Among these receptors, TLR4 stood out as one of the most abundantly expressed and extensively studied receptors in myocardial injury (28). MyD88, an adaptor protein, constitutes a vital element of the TLR4 signaling pathway. Upon TLR4 activation, MyD88 associates with TLR4, subsequently initiating downstream signaling pathways such as NF-κB and MAPK, thereby instigating inflammatory responses and modifications in cellular function (29). The potential of the TLR4/MyD88 signaling axis as a preventive or therapeutic strategy for HFpAMI is increasingly gaining attention. Subsequently, in our cohort, we conducted an exploration of the TLR4/MyD88 signaling axis in the pathological process of HFpAMI using RT-qPCR methods, revealing significantly elevated expression levels of TLR4 and MYD88 in PBMC samples from the HFpAMI group compared to the non-HF group. We hypothesized that these dysregulated genes related to PBMCs may contribute to the pathogenesis of HFpAMI by mediating the TLR4/MyD88 signaling axis.

Recent research has increasingly emphasized the pivotal role of immune cell infiltration in the development and progression of heart failure subsequent to myocardial infarction (7). The increase in neutrophils and decrease in CD4T lymphocytes have been demonstrated to be correlated with the mortality rate among AMI patients (30). In our investigation, we utilized the CIBERSORT algorithm to confirm the link between heightened peripheral neutrophils and reduced peripheral CD4T cells with the progression of HFpAMI, consistent with previous research, thereby validating the reliability of CIBERSORT. Previous research has suggested the correlation between white blood cell count and its subtypes with the progression of AMI. During the acute phase, increased white blood cells are frequently concurrent with AMI, correlating with the degree of necrosis, coronary artery inflammation, and systemic inflammation. Specifically, immediate neutrophil activation occurs post-AMI, with neutrophils being the primary leukocytes detected in infarcted myocardium (31). Given that HF is a severe AMI complication, timely and accurate prediction of HFpAMI is crucial. Arlier studies have demonstrated an independent positive association between increased neutrophil count and larger infarction area, mechanical complications, and mortality rates in AMI patients (32). The role of CD4T cells in HFpAMI is a current research focus. Previous studies have confirmed that a reduction in lymphocytes is a common phenomenon in the acute phase of AMI, especially the decrease in CD4T cell count closely associated with AMI (33). Additionally, the reduction in lymphocytes and specific CD4 counts is correlated with a low ejection fraction, high degree of myocardial necrosis, and mortality rate in AMI patients (34). In this study, we verified through clinical samples that HFpAMI patients exhibited a significant decrease in CD4T cell concentration compared to the non-heart failure patients, accompanied by an increase in neutrophils counts. These findings suggested that the influence of HFpAMI on peripheral immune cells may be more substantial than previously assumed, necessitating further research to elucidate the potential role of these immune cells in HFpAMI.

Macrophages play a critical role in myocardial tissue injury and repair processes, being involved in the complete process of ventricular remodeling after AMI, exerting significant regulatory control from the initial phase of inflammation to the fibrotic remodeling stage (35). Macrophages initially exhibit a pro-inflammatory M1 phenotype, succeeded by an anti-inflammatory M2 phenotype, with these phenotypes demonstrating a time-dependent pattern and exerting distinct or even contradictory roles in various post-AMI stages. During the fibrotic repair phase and stable proliferation phase following AMI, prevailing anti-inflammatory M2 macrophages counteract M1 macrophages, releasing anti-inflammatory, pro-angiogenic, and reparative factors. They actively engage in generating myocardial fibrosis and facilitating tissue repair (36). For instance, in IL-13-knockout myocardial infarction mice, a reduction in M2 macrophages resulted in increased myocardial fibrosis and worsened heart function (37). Regrettably, within our dataset and clinical cohort, we did not identify any distinct differences in M1 and M2 macrophages between HFpAMI and non-HF PBMC samples. As we further explored the relationship between three potential diagnostic biomarkers and immune cells, our investigations revealed a significant negative correlation between CLU, FOS, CXCL8 in PBMC samples from HFpAMI patients and the concentration of M2 macrophages, along with a significant positive correlation with neutrophil count. Consequently, we suggested that CLU, FOS, CXCL8 might contribute to the onset and progression of HFpAMI by modulating various immune cells. However, these hypotheses required further research to unravel the intricate interplay between genes and immune cells.

This study has several limitations. Firstly, the limited number of samples in the validation cohort and the absence of follow-up information might introduce bias in the results and limit the diagnostic ability of gene detection for prognosis. Secondly, although certain immune cell dysfunctions were identified in line with previous studies, the specific roles of these immune cells and the underlying molecular events during the progression of HFpAMI remain insufficiently characterized. Future studies will aim to elucidate the dynamics of immune cell populations, such as macrophages, by integrating spatial transcriptomics or cardiac tissue-specific analyses. Thirdly, CIBERSORT deconvolution relies on the LM22 reference matrix, which may not fully resolve rare PBMC subsets (e.g., plasmacytoid dendritic cells) or account for activation state heterogeneity; this could affect immune infiltration precision despite our stringent quality control. Fourthly, although we linked CLU/FOS/CXCL8 to neutrophil infiltration and M2 macrophage reduction, their precise mechanistic roles in HFpAMI progression, such as whether CLU/FOS/CXCL8 directly drives neutrophil recruitment or indirectly modulates inflammation, remain unresolved. Finally, the PBMCs-related DEGs were solely confirmed in clinical samples without demonstration of their potential functions in cellular or animal models of HFpAMI. These limitations highlight the need for multicenter cohorts, mechanistic experiments, and head-to-head comparisons with existing biomarkers to establish clinical superiority.

## 5 Conclusions

In this study, we have successfully identified three PBMCs-related feature genes (CLU, FOS, CXCL8) using comprehensive bioinformatics analysis and machine learning algorithms. These genes have shown a potential to serve as diagnostic biomarkers for HFpAMI patients. Furthermore, we noticed that naive CD4T cells, and neutrophil may be correlated with the occurrence and progression of HFpAMI, while a significant correlation between CLU, FOS, CXCL8 and M2 macrophages, neutrophil count. Overall, our findings may provide new insights into the pathogenesis and diagnosis of HFpAMI. Further *in vitro* and *in vivo* studies are imperative to elucidate the potential mechanisms of these pivotal genes in HFpAMI.

## Data Availability

The datasets presented in this study can be found in online repositories. The names of the repository/repositories and accession number(s) can be found in the article/Supplementary Material.
