# Supplementary material for: CLU, FOS, and CXCL8 as diagnostic biomarkers for heart failure progression post-acute myocardial infarction: an integrated RNA-Seq and multi-machine learning study
Source: Front Cardiovasc Med. 2025 Jun 30;12:1611668. doi: 10.3389/fcvm.2025.1611668 (PMC12257312; doi:10.3389/fcvm.2025.1611668)
Supplement: Supplementary file 1 [file Datasheet1.docx]

Table S1: The 27 differentially expressed PBMCs-related genes in HFpAMI samples compared to non-HF samples.

| ID | logFC | AveExpr | t | P-Value | adj.P.Val | B |
| --- | --- | --- | --- | --- | --- | --- |
| SNORA22 | 0.774447 | 10.19738 | 6.839074 | 3.00E-09 | 5.65E-05 | 10.81025 |
| FADS2 | 0.928386 | 8.936966 | 5.704797 | 2.91E-07 | 0.00034 | 6.578913 |
| LRRN3 | 0.942636 | 8.122559 | 5.031788 | 3.92E-06 | 0.001011 | 4.175281 |
| PVALB | -0.6744 | 6.882502 | -4.75354 | 1.11E-05 | 0.001677 | 3.217239 |
| CLEC5A | -0.61573 | 6.315095 | -4.68308 | 1.44E-05 | 0.001828 | 2.978581 |
| AK5 | 0.682964 | 7.187278 | 4.605273 | 1.91E-05 | 0.00216 | 2.717045 |
| GPR15 | 1.181533 | 9.237651 | 4.572011 | 2.15E-05 | 0.002309 | 2.605885 |
| FOS | -0.7055 | 11.30727 | -4.42654 | 3.64E-05 | 0.003006 | 2.124525 |
| CD24 | 0.682313 | 6.06593 | 4.187283 | 8.46E-05 | 0.004763 | 1.350844 |
| TAS2R43 | 0.95556 | 5.563776 | 3.85218 | 0.000265 | 0.00854 | 0.308831 |
| ZFP57 | -0.64512 | 6.808102 | -3.73283 | 0.000394 | 0.010611 | -0.04946 |
| CXCL8 | -0.63979 | 7.315995 | -3.70824 | 0.000427 | 0.01111 | -0.12241 |
| ITGA2B | -0.65354 | 8.878167 | -3.41301 | 0.001097 | 0.019596 | -0.97317 |
| CLU | -0.60528 | 9.459894 | -3.35863 | 0.001298 | 0.021734 | -1.12464 |
| SLED1 | -0.61811 | 7.918277 | -3.35495 | 0.001313 | 0.021832 | -1.13485 |
| TRGV5 | -0.62078 | 8.044574 | -3.34753 | 0.001344 | 0.022181 | -1.15537 |
| TNFAIP6 | -0.58873 | 6.351334 | -2.86424 | 0.005584 | 0.049055 | -2.42035 |
| IL1R2 | -0.63752 | 7.140547 | -2.73071 | 0.008078 | 0.061677 | -2.74344 |
| TMEM176A | -0.83227 | 9.497253 | -2.65498 | 0.00991 | 0.068962 | -2.92124 |
| USP9Y | 0.964839 | 8.04791 | 2.461321 | 0.016438 | 0.092756 | -3.35755 |
| UTY | 1.068188 | 9.302973 | 2.216399 | 0.030081 | 0.134018 | -3.86987 |
| RPS4Y1 | 0.84664 | 8.42367 | 2.136481 | 0.036315 | 0.150224 | -4.02713 |
| DDX3Y | 0.972223 | 8.116941 | 2.135862 | 0.036367 | 0.150309 | -4.02833 |
| TMEM176B | -0.59838 | 8.804578 | -2.12566 | 0.03724 | 0.15221 | -4.04803 |
| TXLNGY | 0.877487 | 8.065048 | 2.103737 | 0.039178 | 0.156869 | -4.09012 |
| KDM5D | 0.805023 | 8.44802 | 2.07216 | 0.042122 | 0.163294 | -4.15006 |
| EIF1AY | 0.749634 | 7.593478 | 2.002371 | 0.049315 | 0.179676 | -4.27972 |

Table S2: Information about 18 nodes in the PPI network.

| Gene name | Uniprot ID | logFC | P-Value | Class |
| --- | --- | --- | --- | --- |
| TMEM176A | Q96HP8 | 0.832272453 | 0.009910266 | up |
| FOS | P01100 | 0.705498983 | 3.64E-05 | up |
| PVALB | P20472 | 0.674404994 | 1.11E-05 | up |
| CXCL8 | P10145 | 0.639791162 | 0.00042706 | up |
| IL1R2 | P27930 | 0.637524609 | 0.008078145 | up |
| CLU | P10909 | 0.605275513 | 0.001298425 | up |
| TMEM176B | Q3YBM2 | 0.598380531 | 0.03723984 | up |
| TNFAIP6 | P98066 | 0.588734184 | 0.005584385 | up |
| AK5 | Q9Y6K8 | -0.682964285 | 1.91E-05 | down |
| CD24 | P25063 | -0.682313109 | 8.46E-05 | down |
| EIF1AY | O14602 | -0.749633812 | 0.049315324 | down |
| KDM5D | Q9BY66 | -0.805023381 | 0.042122039 | down |
| RPS4Y1 | [P22090](https://www.uniprot.org/uniprotkb/P22090/entry) | -0.84663999 | 0.036314894 | down |
| LRRN3 | [Q9H3W5](https://www.uniprot.org/uniprotkb/Q9H3W5/entry) | -0.942636061 | 3.92E-06 | down |
| USP9Y | [O00507](https://www.uniprot.org/uniprotkb/O00507/entry) | -0.964838872 | 0.016437732 | down |
| DDX3Y | [O15523](https://www.uniprot.org/uniprotkb/O15523/entry) | -0.972223273 | 0.03636733 | down |
| UTY | [O14607](https://www.uniprot.org/uniprotkb/O14607/entry) | -1.068187823 | 0.030081226 | down |
| GPR15 | [P49685](https://www.uniprot.org/uniprotkb/P49685/entry) | -1.181533 | 2.15E-05 | down |
